# A Convenient In Situ Preparation of Cu_2_ZnSnS_4_–Anatase Hybrid Nanocomposite for Photocatalysis/Photoelectrochemical Water-Splitting Hydrogen Production

**DOI:** 10.3390/molecules29112514

**Published:** 2024-05-26

**Authors:** Ke-Xian Li, Cai-Hong Li, Hao-Yan Shi, Rui Chen, Ao-Sheng She, Yang Yang, Xia Jiang, Yan-Xin Chen, Can-Zhong Lu

**Affiliations:** 1 State Key Laboratory of Structural Chemistry, Fujian Science & Technology Innovation Laboratory for Optoelectronic Information of China, Fujian Institute of Research on the Structure of Matter, Chinese Academy of Sciences, Fuzhou 350108, China; xmlikexian@fjirsm.ac.cn (K.-X.L.); xmlicaihong@fjirsm.ac.cn (C.-H.L.); xmshihaoyan@fjirsm.ac.cn (H.-Y.S.); xmchenrui@fjirsm.ac.cn (R.C.); xmsheaosheng@fjirsm.ac.cn (A.-S.S.); xmyangyang@fjirsm.ac.cn (Y.Y.); xmjiangxia@fjirsm.ac.cn (X.J.); 2 College of Chemistry and Materials Science, Fujian Normal University, Fuzhou 350108, China; 3 Xiamen Key Laboratory of Rare Earth Photoelectric Functional Materials, Xiamen Institute of Rare-Earth Materials, Haixi Institutes, Chinese Academy of Sciences, Xiamen 361021, China; 4 College of Chemistry, Fuzhou University, Fuzhou 350116, China

**Keywords:** copper-based sulfides, heterojunction, photocatalytic H_2_ generation, Cu_2_ZnSnS_4_–anatase nanocomposite

## Abstract

This study details the rational design and synthesis of Cu_2_ZnSnS_4_ (CZTS)-doped anatase (A) heterostructures, utilizing earth-abundant elements to enhance the efficiency of solar-driven water splitting. A one-step hydrothermal method was employed to fabricate a series of CZTS–A heterojunctions. As the concentration of titanium dioxide (TiO_2_) varied, the morphology of CZTS shifted from floral patterns to sheet-like structures. The resulting CZTS–A heterostructures underwent comprehensive characterization through photoelectrochemical response assessments, optical measurements, and electrochemical impedance spectroscopy analyses. Detailed photoelectrochemical (PEC) investigations demonstrated notable enhancements in photocurrent density and incident photon-to-electron conversion efficiency (IPCE). Compared to pure anatase electrodes, the optimized CZTS–A heterostructures exhibited a seven-fold increase in photocurrent density and reached a hydrogen production efficiency of 1.1%. Additionally, the maximum H_2_ production rate from these heterostructures was 11-times greater than that of pure anatase and 250-times higher than the original CZTS after 2 h of irradiation. These results underscore the enhanced PEC performance of CZTS–A heterostructures, highlighting their potential as highly efficient materials for solar water splitting. Integrating Cu_2_ZnSnS_4_ nanoparticles (NPs) within TiO_2_ (anatase) heterostructures implied new avenues for developing earth-abundant and cost-effective photocatalytic systems for renewable energy applications.

## 1. Introduction

The quest for clean energy solutions has become imperative to address the escalating environmental pollution and energy shortages. The rampant release of nitrogen, sulfur oxides, and other emissions from fossil fuel combustion, including greenhouse gas CO_2_, has significantly tainted our environment. Considering this, hydrogen energy has garnered substantial interest in recent years due to its environmental sustainability, high energy density by weight (approximately 143 MJ/kg calorific value), and potential for recyclability [1,2]. The concept of photocatalytic water splitting, a promising avenue for green hydrogen production, was first demonstrated by Fujishima and Honda in 1972. This process efficiently converts sunlight into hydrogen, making it an ideal technology for sustainable energy production [3]. However, effective photocatalysts for the hydrogen evolution reaction (HER) must satisfy several requirements: (i) the photo-excited valence band (VB) holes must have an oxidation-reduction potential that is sufficiently positive to serve as electron acceptors, and (ii) the photo-excited electrons in the conduction band (CB) should exhibit an oxidation-reduction potential that is more negative than that of the H_2_/reduction product redox potential [4]. Given these prerequisites, photocatalysts that feature optimal band gaps (ranging from 1.45 to 3.3 eV) and can harness approximately 43% of the solar spectrum are known to exhibit robust redox capabilities essential for photocatalytic HER. Therefore, identifying suitable semiconductor materials has become a critical endeavor [5,6].

In the field of semiconductor photocatalysis, exploiting semiconductor materials like g-C_3_N_4_, CdS, and Cu-based sulfides for photocatalytic water splitting has been recognized as a promising avenue for hydrogen production, addressing the global energy challenge [6]. TiO_2_ has emerged as a promising photocatalyst, celebrated for its exceptional stability and resistance to photo and chemical corrosion during reactions. Its suitable band gap allows for excitation by ultraviolet light, while its high catalytic activity and photo-generated charge carriers with a strong redox capacity have garnered significant scientific interest. This surge in attention traces back to the groundbreaking work by Fujishima and Honda, who first reported on photoelectrochemical (PEC) water splitting using TiO_2_ electrodes [4,7,8,9,10,11]. However, the practical application of TiO_2_ is somewhat constrained by its relatively large band gap (3.0~3.2 eV) and the rapid recombination rate of light-generated electron–hole pairs, limiting its efficient utilization of visible light [7,12]. Various strategies have been explored to overcome these limitations, including elemental doping, surface modification, and semiconductor coupling with materials like CdS and CeO [1,12,13]. It has been discovered that anchoring elements and forming heterojunctions with p-type semiconductors can effectively narrow the band gap and broaden the spectral response range of n-type TiO_2_. These methods serve as cost-effective and performance-enhancing strategies to amplify TiO_2_’s photocatalytic efficiency. Specifically, the transfer of interfacial electrons from the n-type to the p-type region plays a pivotal role in generating photoluminescent electron–hole pairs, promoting their separation, and prolonging their lifetime [4,11,14,15].

The quaternary semiconductor Cu_2_ZnSnS_4_ (CZTS) has emerged as a standout non-toxic absorber material in the quest for environmentally friendly and cost-effective photocatalysts. Owing to its direct band gap of 1.50 eV, high absorption coefficient (~10^4^ cm^−1^), substantial crustal abundance (Cu: 55 ppm, Zn: 80~100 ppm, Sn: 2.2 ppm), and robust stability, CZTS has become a promising candidate material [2,8,14,16,17,18,19,20,21,22]. Its appealing properties have led to its widespread adoption in heterojunction coupling applications. Historically, Cu-sulfur-based materials like Cu(In, Ga) (S, Se)_2_ (CIGS) and Cu_2_ZnSnS_4_ (CZTS) have undergone extensive investigation. Specifically, CZTS-based p-n heterojunction photocatalysts have been explored—including configurations such as CZTS/ZnO, CZTS/ SnO_2_, CZTS/WO, and CZTS/La_2_TiO_7_, which have been successfully utilized for the degradation of organic pollutants and CO_2_ reduction. These studies underscore CZTS’s efficacy as a suitable co-catalyst in p-n heterojunction photocatalytic applications [1,4,7,12,13,21].

CZTS has emerged as a highly effective metal semiconductor for producing thin film photo-cathodes in PEC (photoelectrochemical) water-splitting cells, focusing on catalyzing the hydrogen evolution reaction (HER). Sami and his team utilized pulsed laser deposition (PLD) techniques to fabricate Cu_2_ZnSnS_4_ (CZTS) films with varying thicknesses on soda-lime glass substrates. Their investigation revealed that larger CZTS particles could hinder light propagation, thus impacting the film’s ability to absorb photons effectively. Notably, a CZTS photocatalyst with a thickness of 242 nm exhibited the highest photocatalytic performance among all of the samples tested [23]. These findings illustrate how manipulating nanocrystal size and other synthesis parameters can finely adjust the material’s physical and optical characteristics. CZTS displays enhanced photocatalytic activity at the nanoscale level compared to its bulk form, making it an excellent choice for applications such as counter electrodes and photocathodes in solar cells [24]. Jiang and his team also reported significant progress by employing a spray-sulfurization method for CZTS absorber layers, combined with a sandwich-structured buffer layer of HfO_2_/CdS/HfO_2_. This configuration achieved a remarkable photocurrent density (Jph) of 28 mA cm^−2^ at 0 V_RHE_ (voltage vs. reversible hydrogen electrode) and an onset potential (Von) of 0.72 V_RHE_, resulting in an impressive hydrogen conversion efficiency of 7.27% [25]. In a related study, Liang and his colleagues developed CZTSSe (Cu_2_ZnSn(S,Se)_4_) light-absorbing films characterized by high crystallinity and a dense crystal structure. They constructed a Mo/CZTSSe/CdS/TiO_2_/Pt photocathode, focusing on optimizing the thickness of CZTSSe to minimize defect density and maximize built-in voltage. This optimized configuration significantly reduced defect-assisted recombination, thereby improving charge transfer and separation efficiencies. Notably, the half-cell solar hydrogen production efficiency of the optimized CZTSSe photocathode reached 6.47% [26,27]. These accomplishments underscore the promising potential of modified CZTS materials for efficient solar hydrogen production, highlighting the significant role CZTS-based materials could play in enhancing photocathode performance for solar-powered hydrogen generation.

The above study demonstrates how p-n junctions can be effectively tuned by adjusting the composition to modulate band gap energy levels, creating a band structure optimized for water reduction, and achieving a relatively high photocatalytic and PEC performance [14,28]. Motivated by the remarkable properties of CZTS, there is anticipation that p-n heterostructures comprising CZTS–TiO_2_ nanocomposite photocatalysts could serve as viable, precious-metal-free, and environmentally friendly catalysts in photocatalytic hydrogen evolution reactions.

This paper presents a simple in situ method for synthesizing a CZTS–anatase hybrid nanocomposite. This approach reduces the band gap of titanium dioxide and enhances the concentration of photo-generated charge carriers, significantly improving the photocatalytic performance for hydrogen evolution. Extensive studies were conducted on the microstructure and photoactivity of the CZTS–anatase composite materials. The results indicate that the photocatalytic activity of the CZTS–anatase heterojunction-structured nanocomposite is enhanced by the formation of p-n heterojunctions with CZTS acting as a co-catalyst, surpassing the performance of both pristine CZTS and anatase alone. Furthermore, this cost-effective, robust, and versatile method allows for synthesizing multiphase/TiO_2_ heterojunction semiconductors in a relatively short time and at lower temperatures, offering a promising avenue for developing advanced photocatalytic materials.

## 2. Results and Discussion

### 2.1. Structure and Morphology

**Figure 1:**
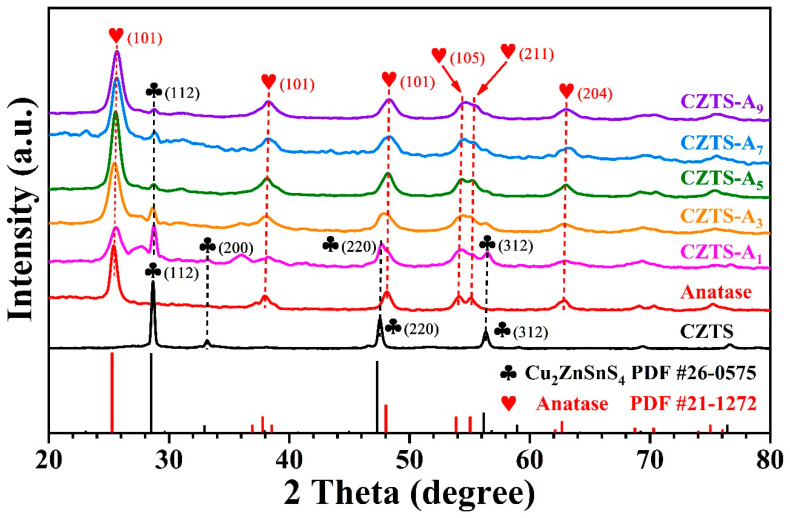
XRD patterns of the Cu_2_SnZnS_4_ (CZTS), the anatase TiO_2_, and the CZTS–A_X_ (X = 1, 3, 5, 7, 9).

The crystalline phases and structural characteristics of the synthesized CZTS NPs, anatase titanium dioxide synthesized by TOBT, and hybrid CZTS–A_X_ (X = 1, 3, 5, 7, 9) nanocomposites were analyzed using X-ray diffraction (XRD). As depicted in Figure 1, the X-ray diffraction spectra present characteristic peaks at 2θ = 28.5°, 47.3°, and 56.1°, which correspond to the (112), (220), and (312) facets of Kesterite CZTS (JCPDS 26-0575) [17,29,30,31], respectively. The lattice constants derived from the XRD peaks are a = 0.542 nm and c = 1.093 nm. The XRD spectra of the CZTS–Ax (X = 1, 3, 5, 7, 9) nanocomposites prepared by TBOT are at 2θ = 25.3°, 37.8°, 48.0°, 53.9°, 55.0°, 62.7°, and 68.7°, respectively, corresponding to the (101), (004), (200), (105), (211), (204), and (116) planes, which could be associated with anatase TiO_2_ (JCPDS 21-1272). All the mixed CZTS–Ax samples exhibited characteristic peaks of anatase titanium dioxide, and potassium feldspar peaks are also observed at 2θ = 28.4°, 47.3°, and 56.1°, indicating the successful hybridization of the two. With the increase in the addition of anatase (TBOT was added to the precursor solution in varying proportions) to 9 mL, the XRD apex of anatase at 2θ = 25.3°, 48.0° is more distinct, suggesting the successful hybridization of anatase and potassium feldspar CZTS composites. By observing the XRD spectra and diffraction patterns, it can be discerned that the sharpness of the peaks in the patterns has decreased, indicating that they are nanocrystalline, and the level of crystallinity decreases accompanied by the addition of TBOT. In the synthesis process exemplified by CZTS–A_5_, the molar ratio of CZTS to TiO_2_ was maintained at 1:3, which resulted in the suppression of CZTS peaks due to the addition of titanium dioxide. The typical grain size of each sample was calculated using the Debye–Scherrer equation (see Equation (1)) [30].

(1) $$d=\frac{0.9\times \lambda }{\beta \times cos\theta }$$

Based on the comprehensive XRD spectrum analysis results, it can be inferred that the grain size of the rutile TiO_2_ and CZTS composite material slightly decreases, and the crystallinity decreases. These changes may have a significant impact on the photocatalytic performance of the material. The reduced grain size may increase the surface area of the material, increase the density of active sites in photocatalytic reactions, and thus promote the generation and separation of photo-generated electron–hole pairs. However, the decrease in crystallinity may lead to an increase in lattice defects, affecting the efficiency of electron transfer and photocatalytic reactions. Therefore, the grain size and crystallinity changes in rutile TiO_2_ and CZTS composite materials may jointly affect their photocatalytic performance, and further experimental verification and in-depth research are needed to evaluate their effects comprehensively.

**Figure 2:**
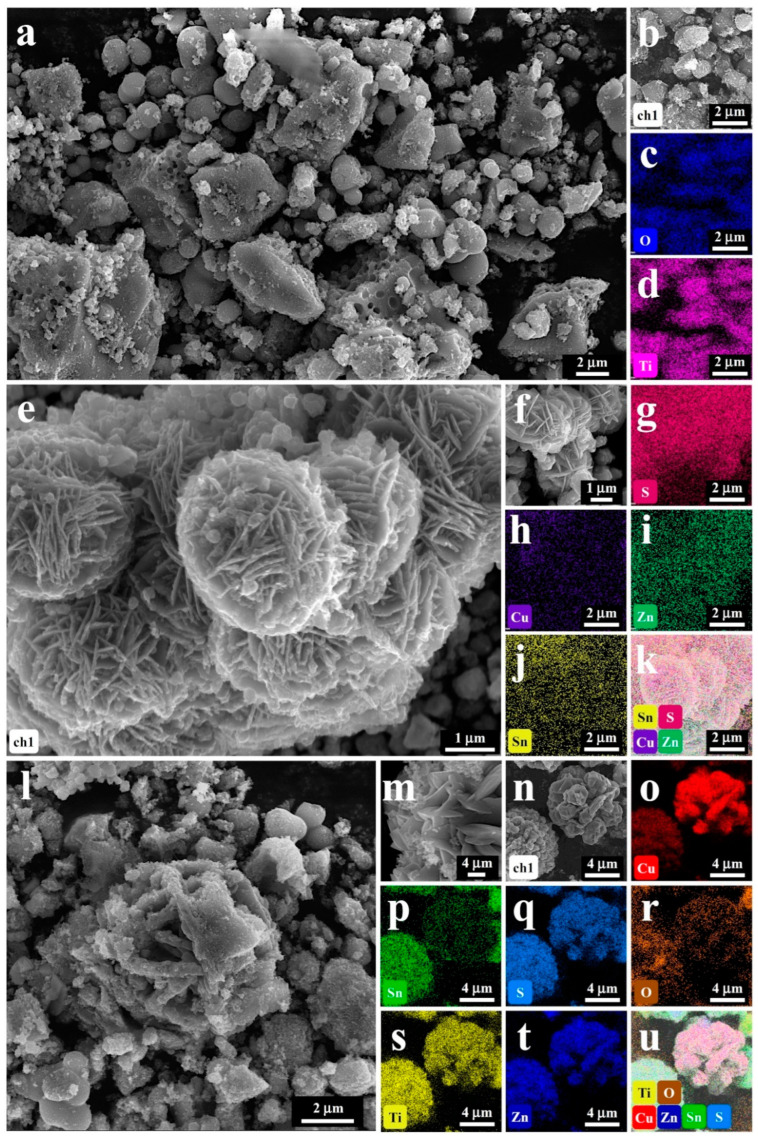
SEM and EDS-mapping images (Cu, Zn, S, Sn, O, and Ti) of (**a**–**d**) anatase; (**e**–**k**) CZTS; (**l**–**u**) CZTS–A_5_.

The morphology and microstructure characteristics of composite materials were extensively studied using scanning electron microscopy (SEM) and high-resolution transmission electron microscopy (HR-TEM). Figure 2 shows the SEM and EDS mapping images of the anatase materials (Figure 2a–d), CZTS materials (Figure 2e–k), and CZTS–A_5_ nanocomposite materials (Figure 2l–u), which were obtained by hydrothermal methods. Figure 2a shows that the anatase material exhibits a typical spherical particulate structure, Figure 2c,d shows that the Ti and O elements are uniformly distributed in the EDS, Figure 2e shows that the CZTS is assembled in nanosheets, and the EDS spectra (Figure 2g–j) show a uniform distribution of the elements. In addition, the SEM images of the CZTS nanoparticles, obtained from the precursor without surfactants and the tetra-n-butyl titanate (TBOT), reveal a relatively homogenous distribution of CZTS nanoparticles exhibiting a flower-like structure and nanosheet morphology (refer to Figure 2l–n). However, introducing TBOT leads to dense agglomerations, presumably due to increased surface energy, which promotes compact clustering and diminishes crystallinity. A comparative analysis of Figure 2 with Figures S1 and S2 indicates that all hybrid composites display similar morphologies, featuring Kesterite CZTS and TiO_2_ (anatase) heterojunction structures.

Nonetheless, incorporating TBOT initiates a gradual transition towards larger TiO_2_ particle-like morphologies, accompanied by a reduction in nanosheet forms. As the TiO_2_ concentration escalates, the nanosheet particles progressively evolve into an average particle size ranging from 20 to 40 nm. The XRD results indicate that the average grain size of the hybrid nanocomposite materials (40 nm) is significantly smaller than depicted in the SEM images. This suggests that larger particles are possible because of TiO_2_ (anatase) accumulation and the aggregation of small CZTS nanograins on the TiO_2_ (anatase) surface. Furthermore, the identification cannot distinguish TiO_2_ (anatase) and CZTS nanocrystals.

An EDS spectrum element analysis was performed to further analyze the elemental distribution of the CZTS–A_5_ nanocomposite material. The EDS spectrum analysis in Figure 2c–h indicates that the identified elements in the composite material mainly consist of (Ti), (O), (Cu), (Zn), (Sn), and (S). The elemental mapping indicates that the Cu, Zn, Sn, and S elements are uniformly clustered together, while the Ti and O elements are encapsulated on the periphery, providing data for the successful synthesis and hybridization of the TiO_2_ (anatase) and CZTS composite materials.

**Figure 3:**
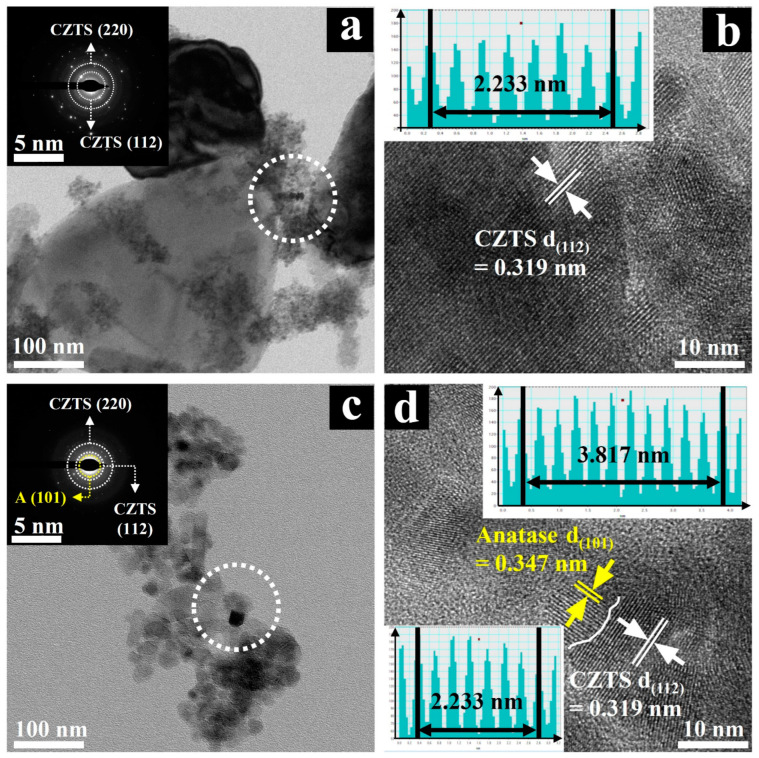
The TEM/HRTEM images and the corresponding SAED patterns of (**a**,**b**) CZTS and (**c**,**d**) CZTS–A_5_.

Additionally, as shown in Figure 3a,c, high-resolution images generated from TEM analysis illustrate the hydrothermally synthesized CZTS NPs and the heterogeneous hybrid CZTS–A_5_ nanocomposite materials. The images reveal that the CZTS sample consists of nanosheet structures with an average diameter of approximately 40 nm.

Furthermore, the selected area electron diffraction (SAED) image, which was recorded from the dotted circle area of Figure 3a, shows that the synthesized CZTS NPs exhibit polycrystalline properties, featuring distinctive rings corresponding to CZTS (112)/(220) planes, consistent with XRD results. In addition, the lattice fringe and IFFT image of CZTS, as shown in Figure 3b, clearly indicate a lattice spacing of 0.319 nm related to the CZTS (112) crystal plane, which is in good agreement with the literature [14,18,32,33].

The SAED pattern of the CZTS–A_5_ sample, recorded from the dotted circle area in Figure 3c, shows featuring distinctive rings corresponding to CZTS (220)/(112) and anatase (101) planes. A representative lattice fringe and IFFT image of the CZTS–A_5_ sample are demonstrated in Figure 3d. The lattice spacing of 0.342 nm is consistent with the (101) plane of TiO_2_ (anatase), and 0.319 nm aligns with the (112) plane of kesterite CZTS. The HRTEM image of CZTS–A_5_ exhibits the uniform distribution of nanoscale TiO_2_ nanoparticles on the sheet-like CZTS structure, with a complete interface contact between TiO_2_ (anatase) and CZTS evident (white curve), forming effective embedding. Which further confirms the formation of heterogeneous structures.

### 2.2. XPS Characterization

**Figure 4:**
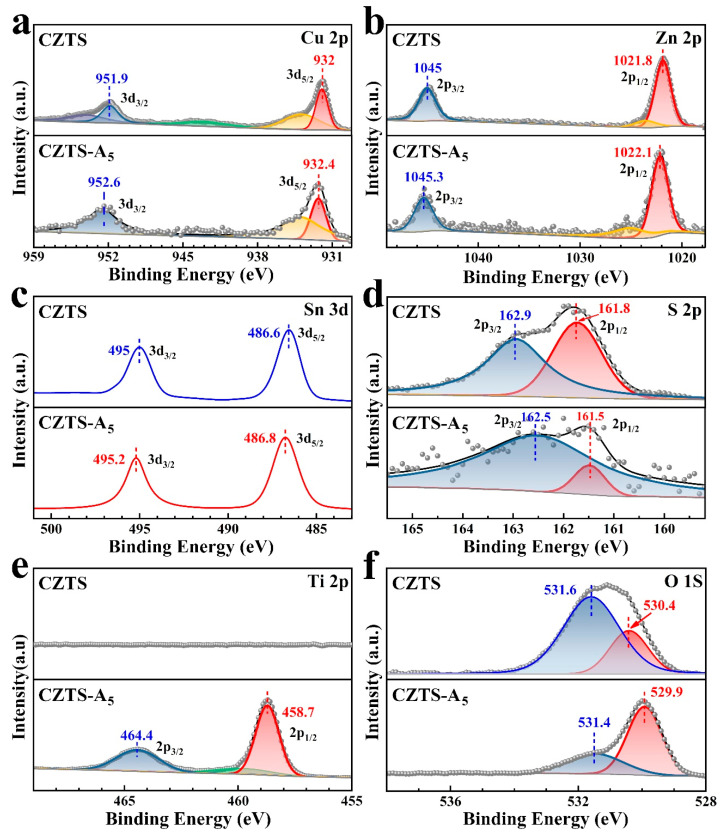
XPS spectra of the CZTS NPs and the CZTS–A_5_ nanocomposites, which involve (**a**) Cu 3d, (**b**) Zn 2p, (**c**) Sn 3d, (**d**) S 2p, (**e**) Ti 2p, and (**f**) O 1s.

Advanced X-ray photoelectron spectroscopy (XPS) was used to analyze the CZTS–A_X_ nanocomposites to determine the composition of the major elements in the CZTS–A_X_ (X = 5) nanocomposites as well as their electronic states (Figure 4 and Figure S3). All elemental analytical energy spectra were calibrated concerning the binding energy (BE) of the 284.6 eV (C 1s) peak for this analysis. As shown in Figure 4a, by analyzing the valence band spectra of each element, in the Cu 2p spectrum, the two apexes of CZTS were clustered at 932 eV (Cu 2p_1/2_) and 951.9 eV (Cu 2p_3/2_), showing a peak distinction of 19.9 eV, which corresponds to Cu 2p, while the binding energies of Cu 2p were concentrated in CZTS–A_X_ at 932.4 eV (Cu 2p_1/2_) and 952.6 eV (Cu 2p_3/2_), showing a binding energy difference of 20.2 eV. These results confirm the presence of Cu^+^ in both cases. Two accompanying satellite peaks were observed at binding energies of 934.0 eV, 954.1 eV, and 943.4 eV. These peaks indicate the presence of Cu^2+^ valence states in the sample due to an increase in copper content, which may lead to adjustments in the electronic structure of the material, thereby affecting its photocatalytic performance. Specifically, the presence of Cu^2+^ may alter the material’s light absorption characteristics, electron transfer performance, and distribution of surface active sites, thereby affecting the rate and efficiency of photocatalytic reactions [14,32,33,34].

XPS spectra also revealed the Zn 2p binding energy peaks, with three distinct peaks observed in Figure 4b. In CZTS, two strong peaks are located around 1045.0 eV (Zn 2p_1/2_) and 1021.8 eV (Zn 2p_3/2_), separated by 23.2 eV. In CZTS–A_5_, the intense peak is concentrated at 1045.3 eV (Zn 2p_1/2_) and 1022.1 eV (Zn 2p_3/2_), maintaining the same splitting value of 23.2 eV, indicating the presence of the Zn^2+^ oxidation state [32,33,35]. The appearance of Zn^2+^ may be related to the conductivity and light absorption characteristics of the material. In Figure 4c, two satellite peaks of the Sn 3d binding energy peak appear. In CZTS, the Sn binding energy is concentrated near 495.0 eV (Sn 3d_3/2_) and 486.6 eV (Sn 2d_5/2_), while in CZTS–A_5_, it is concentrated at 486.8 eV (Sn 2d_5/2_) and 495.2 eV (Sn 3d_3/2_). The regular splitting is measured to be 8.6 eV, confirming the oxidation state of Sn^4+^. The presence of Sn^4+^ may alter the charge transfer characteristics and surface reactivity of the material, thereby affecting its photocatalytic performance. In Figure 4d, the S 2p shows two distinct peaks with a binding energy disparity of 1.1 eV, positioned at 162.9 eV (S 2p_1/2_) and 161.8 eV (S 2p_3/2_). This range is consistent with the reported binding energy of sulfur in sulfides, indicating the oxidation state of sulfides. The binding energy shift of the S 2p peak in CZTS is lower, indicating the electron enrichment of sulfur, suggesting the presence of sulfur vacancies [32,36,37]. These phenomena may affect the surface chemical reactions and photo-generated charge separation processes of materials, affecting their photocatalytic performance. The XPS spectra in Figure 4e also show the Ti 2p binding energy peak. In CZTS–anatase, the Ti binding energies of Ti 2p_1/2_ and Ti 2p_3/2_ are mainly located at 464.4 eV and 458.7 eV, individually, indicating the presence of the Ti^4+^ oxidation state [12,33]. The absence of the Ti peak in CZTS confirms the successful doping of TiO_2_. In addition, Figure 4f shows an O 1s peak centered at 531.6 eV in CZTS, with its intensity decreasing with the addition of TiO_2_. In CZTS–A_5_, the characteristic O 1s peak shifts to 529.9 eV, corresponding to fully oxidized lattice oxygen. The intensity changes, and peak position shifts of the O1s peak also indicate changes in the oxidation state of the material, which may affect the surface chemical activity of the material and the rate of photocatalytic reactions. XPS measurements confirm that all the corresponding peaks in the CZTS–A_5_ nanocomposites are highly consistent with the reported values, indicating no substantial differences regarding the electronic configuration of the respective materials [14,33].

### 2.3. FTIR Characterization and Optical Characterization

**Figure 5:**
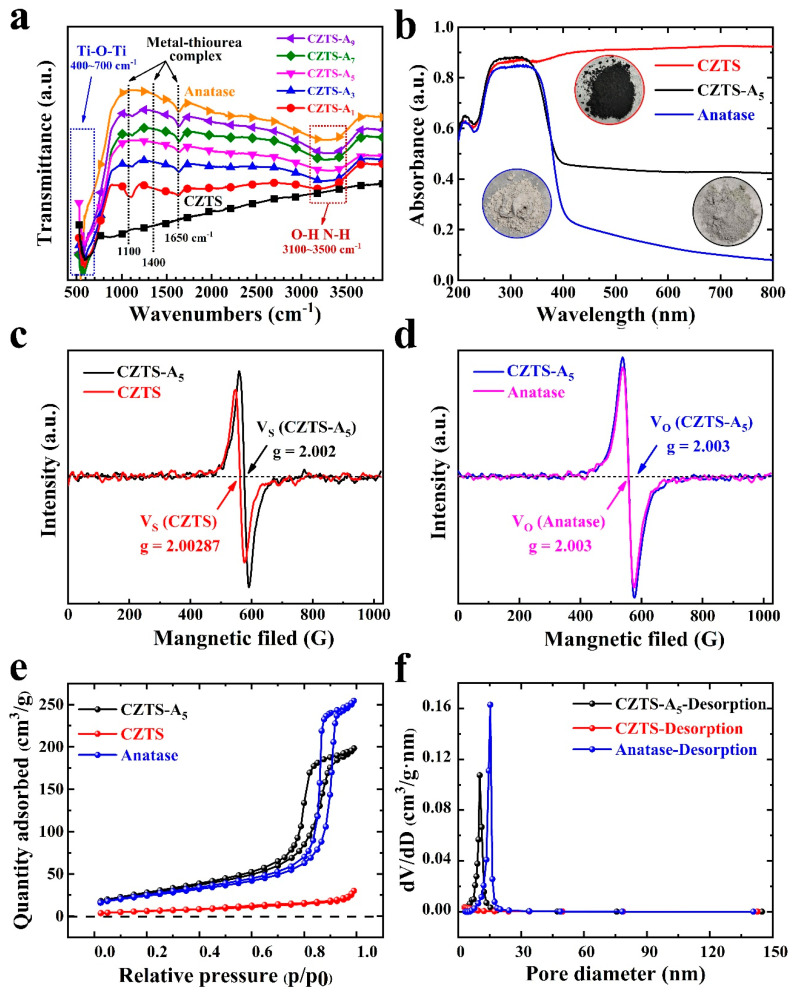
CZTS, TiO_2_ (anatase), and CZTS–A_X_ (X = 1, 3, 5, 7, 9) nanocomposites of FTIR spectra (**a**), CZTS, TiO_2_ (anatase), and CZTS–A_5_ nanocomposites of (**b**) UV–Vis analysis, (**c**,**d**) EPR spectra of CZTS NPs, TiO_2_ (anatase) NPs, and hybrid CZTS–A_5_ nanocomposites recorded at room temperature. (**e**,**f**) N_2_ adsorption–desorption isotherms of CZTS NPs, hybrid CZTS–A_5_ nanocomposites, and TiO_2_ (anatase).

Figure 5a presents the Fourier transform infrared spectra of functional groups in CZTS samples with different doping concentrations of titanium oxides (anatase). The spectra exhibit a broad feature within the range of 3100–3500 cm^−1^, which can be attributed to the typical characteristics of physically adsorbed water and the stretching vibration of thiourea (~3400 cm^−1^) [14,37,38]. In addition, as the concentration of titanium dioxide increases, broad absorption peaks corresponding to the stretching vibration modes of Ti-O and Ti-O-Ti groups appear in the 400–700 cm^−1^ region, and these modes increase with the increase in concentration. Moreover, the main peaks corresponding to the metal–thiourea complex are also observed at around 1100 cm^−1^, 1400 cm^−1^, and ~1650 cm^−1^ [14,39,40]. These values largely coincide with those reported in the research papers, but slight deviations exist. The ultraviolet–visible absorption properties of TiO_2_ (anatase), CZTS NPs, and the mixed CZTS–A_5_ nanocomposite were investigated using a UV–Vis spectrometer, as shown in Figure 5b. The UV–Vis absorption spectra from CZTS NPs recorded within the wavelength range of 200–800 nm show a significant response throughout the visible spectrum, while TiO_2_ (anatase) NPs show a response only below 390 nm [14,37,38]. Furthermore, the absorption of the CZTS–A_5_ nanocomposite sample is enhanced within the wavelength range of 350–800 nm compared to TiO_2_ (anatase), indicating a complementary relationship between the synthesized CZTS and TiO_2_, which correspond to their narrow band gap (1.5 eV) and wide band gap (3.2 eV), respectively [14,16].

The band gap energy (*E_g_*) can be determined by analyzing UV–visible diffuse reflectance spectroscopy (UV–Vis-DRS) data, specifically utilizing the Tauc plot. The formula for calculating *E_g_* is as follows:

(2) $$\alpha hv=A{\times \left(hv-E_{g}\right)}^{\frac{1}{2}}\;$$

In Equation (2), which illustrates the Tauc plot, α signifies the direct light absorption coefficient, h is Planck’s constant, ν represents the frequency of incident light, A is a proportionality constant, and *E_g_* denotes the band gap energy of the semiconductor photocatalyst [35]. The band gap can be estimated based on the Tauc plot (Equation (3)). Consequently, from the test results of UV–visible diffuse reflectance spectroscopy, the band gaps of CZTS, TiO_2_, and CZTS–A_5_ can be inferred to be 1.25, 3.20, and 2.60 eV, respectively (as shown in Figure S4).

Conversely, the hybrid sample CZTS–A_5_ exhibits weaker light absorption compared to CZTS, and its absorption in the visible light region is better than that of TiO_2_ (anatase), indicating the formation of a heterojunction between TiO_2_ (anatase) and CZTS, which covers the entire visible light spectrum. The HRTEM images reveal a good interface combination between TiO_2_ and CZTS, suggesting that a heterojunction can evoke a sufficiently strong electric field, promoting photo-induced charge separation/transfer.

### 2.4. EPR Characterization

As shown in Figure 5c,d, further confirmation of sulfur/oxygen vacancies (in CZTS and CZTS–A_5_) is provided by electron paramagnetic resonance (EPR) spectroscopy. Due to the abundance of unpaired electrons in materials possessing vacancy defects, Electron Paramagnetic Resonance (EPR) is commonly used to identify these unpaired electrons within the material. This, in turn, facilitates the recognition and quantification of vacancies through EPR analysis [41].

From Figure 5c, a signal located at g = 2.00287 and g = 2.002 is detected in CZTS and CZTS–A_5_, respectively, which is attributed to the F center, i.e., a sulfur vacancy carrying a single negative charge [36,41], confirming the presence of sulfur vacancies in CZTS and CZTS–A_5_ prepared using this method.

Additionally, the comparison with Figure 5d reveals that adding TiO_2_ (anatase) leads to the formation of oxygen vacancies in CZTS–A_5_, as indicated by the detection of a signal at g = 2.003, which is precisely the same as that signal from the pure anatase TiO_2_. Upon comparing the peak intensities of CZTS–A_5_ and TiO_2_, a marginal amplification was detected in CZTS–A_5_ relative to TiO_2_. Such an observation can be explained by the augmented number of oxygen vacancies arising from the composition of the hybrid material [42]. This boost possibly originates from the synergistic effect between CZTS–A_5_ and TiO_2_, which presumably catalyzes the creation of oxygen vacancies.

By comparing the peak intensity of sulfur vacancies and oxygen vacancies, it can be deduced that the doping of TiO_2_ (anatase) forms heterojunctions with CZTS, leading to the generation of more defects and, consequently, more vacancies. Oxygen vacancies can reshape the electronic configuration on the material’s exterior, thereby facilitating meticulous manipulation of the density of active electrons and significantly adjusting the semiconductor’s band gap. Furthermore, sulfur (S) vacancies can reduce the energy required for hydrogen adsorption [37,40]. As a result, the proliferation of these vacancies plays a favorable role in augmenting the catalytic proficiency of the CZTS-TiO_2_ (anatase) heterojunction.

### 2.5. BET Characterization

To investigate the influence of the composite on the structure of the composite material among semiconductors, the specific surface area and porosity of the catalyst were comparatively analyzed. The Brunauer-Emmett-Teller (BET) structural properties of Cu_2_ZnSnS_4_ (CZTS), TiO_2_ (anatase), and mixed CZTS–A_X_ (X = 3, 5, 9) nanocomposites were primarily determined via the nitrogen adsorption–desorption isotherm.

As shown in Figure 5e,f, the samples belong to the mesoporous structure, and the isotherms of all the comparative samples exhibit a hysteresis loop characteristic of type IV behavior. As shown in Table 1, the measured BET-specific surface areas are as follows: TiO_2_ (anatase) (87.223 m^2^g^−1^), Cu_2_ZnSnS_4_ (CZTS) (22.022 m^2^g^−1^), and mixed CZTS–A_X_ (X = 3, 5, and 9) (132.280, 98.954, and 126.612 m^2^ g^−1^). Therefore, the hybrid nanocomposites have significantly higher specific surface areas compared to pure TiO_2_ (anatase) and Cu_2_ZnSnS_4_ oxides. Notably, CZTS–A_5_ has the smallest average particle size, indicating a better performance due to increased contact with the sacrificial agent. From Figure S6, we can see that the pore size of CZTS–A_5_ is a bit larger compared to CZTS–A_3_, and it can be considered that its particle size is smaller, but its stacked-up pore size becomes larger, so it leads to a smaller specific surface area. In addition, the addition of TBOT also shows the ability to modulate the textural properties of the sample, such as specific surface area and porosity.

### 2.6. Electrochemical (EC), Photoelectrochemical (PEC) and Photocatalytic (PC) Performance

**Figure 6:**
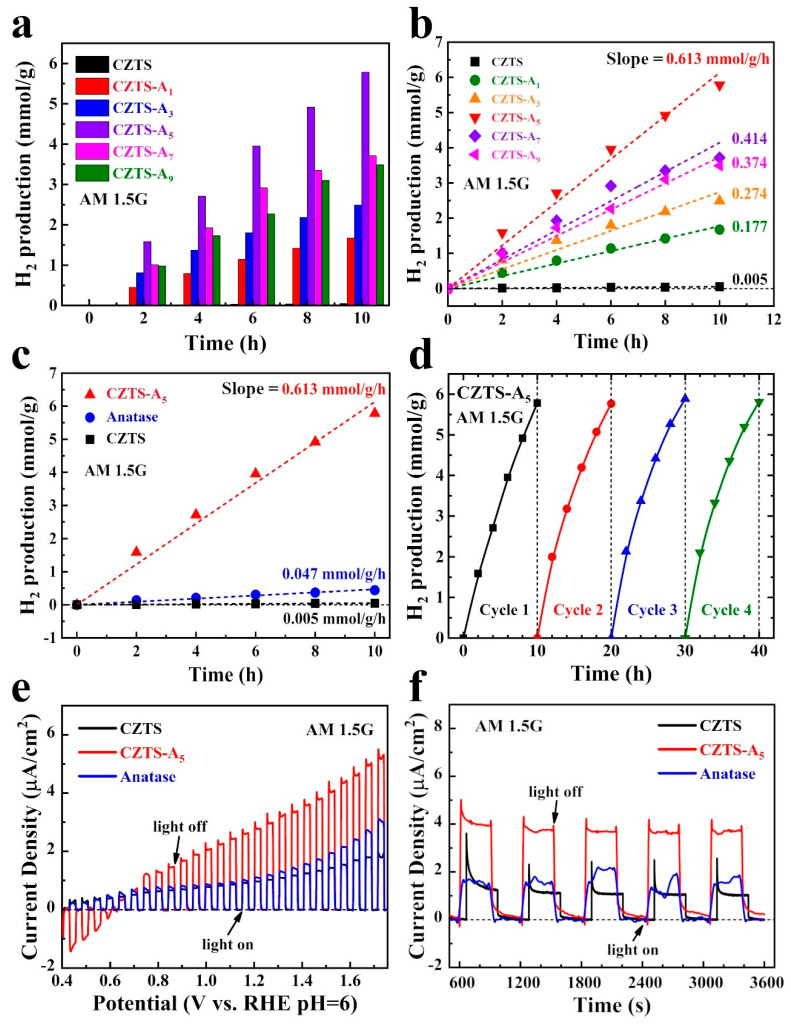
(**a**,**b**) CZTS NPs, hybrid CZTS–A_5_ nanocomposites, and TiO_2_ (anatase) photocatalyzes the water–splitting H_2_ generation for 10 h using ethanol as a sacrificial agent under full spectrum light (the slopes associated with the hydrogen reaction rate simulations are 0.613, 0.414, 0.374, 0.274, and 0.177 mmol/g/h, respectively); (**c**) The photocatalytic activity of CZTS, CZTS–A_5_, and TiO_2_ (anatase) with the existence of ethanol as a sacrificial agent for 10 h; (**d**) Cyclic test of H_2_ generation in CZTS–A_5_ samples under full spectrum light irradiation; (**e**) Chopped linear sweep voltammetry (LSV) curve; and (**f**) chrono amperometry data plot of CZTS, CZTS–A_5_, and TiO_2_ (anatase) samples observed under the bias voltage of 1.5V (vs. RHE, the electrolyte is 0.5 M Na_2_SO_4_, pH = 6).

This study evaluated the photocatalytic hydrogen production performance of CZTS NPs, TiO_2_ (anatase) NPs, and CZTS–A_X_ (X = 1, 3, 5, 7, and 9) nanocomposites. The results are shown in Figure 6a,b.

Figure 6a,b shows that the hydrogen production of pure TiO_2_ (anatase) and CZTS NPs is deficient, which can be attributed to the high recombination rate of photo-generated electrons/holes. Compared with pure TiO_2_ (anatase) and CZTS NPs, the hydrogen production of hybrid CZTS–A_5_ increased by about 11 times (0.0465 vs. 0.5017 mmol/g/h, CZTS–A_5_ vs. TiO_2_ anatase) and about 250 times (0.002 vs. 0.5017 mmol/g/h), respectively. The results indicate that the p-n heterojunctions formed by combining CZTS and TiO_2_ (anatase) promote effective charge transfer and separation between interfaces, reducing the recombination rate of photo-generated electrons/holes.

In addition, as shown in Figure 6c, in order to further understand the comparison of the photocatalytic hydrogen production performance of anatase, CZTS, and composite materials, we compared the photocatalytic performance of anatase, CZTS, and composite CZTS–A_5_ samples. It can be seen that compared with single CZTS and anatase, the composite CZTS–A_5_ can produce hydrogen at a rate of 0.613 mmol/g/h, while CZTS and anatase produce only 0.005 mmol/g/h and 0.047 mmol/g/h. As shown in Figure 6b, the CZTS–A_5_ sample, which has a 5 mL TBOT addition, exhibits the best photocatalytic H_2_ generation performance. To investigate the stability of photocatalysts, we centrifuged the CZTS–A_5_ sample from the electrolyte after ten hours of photocatalysis, refreshed the sacrificial agent, and subjected it to four light cycles. The obtained data are almost unchanged, as shown in Figure 6d.

Further research evaluated the photoelectrochemical (PEC) performance of the as-prepared CZTS–A_X_ samples with different concentrations of TBOT content. Figure 6e shows the linear sweep voltammetry (LSV) curves of the CZTS, CZTS–A_5_, and TiO_2_ (anatases) photoanodes under the AM 1.5G light irradiation.

The photocurrent density of the CZTS–A_5_ sample is 2.81 µA cm^−2^ at 1.23 V relative to RHE, which is 2.6 times that of TiO_2_ (1.08 µA cm^−2^) and about 3 times that of the original CZTS (0.93 µA cm^−2^). The improvement in photoelectric performance can be attributed to the forming of a p/n junction, which enhances the charge separation mechanism, resulting in a strong photocurrent response in CZTS–A_5_; however, as illustrated in Figure S5, the light-induced current behavior in the mixed nanocomposites with a higher TiO_2_ content (such as CZTS–A_7_ and CZTS–A_9_) has decreased, which is due to the dominance of the TiO_2_ (anatase) content, resulting in the formation of a composite center, thereby accelerating the electron–hole recombination, see Figure S5.

We further explored the stability of the photoanode under different specific bias voltages. Typically, the chronoamperometry measurements were conducted at 1.5 V. (vs. RHE pH = 6) to verify the stability of the catalyst, as shown in Figure 6f. Compared to CZTS and TiO_2_, CZTS–A_5_ exhibits the highest photocurrent density, as shown in LSV. Additionally, in terms of stability, the CZTS–A_5_ sample exhibits a higher stability over extended cycles compared to TiO_2_, indicating a significant improvement in photostability.

**Figure 7:**
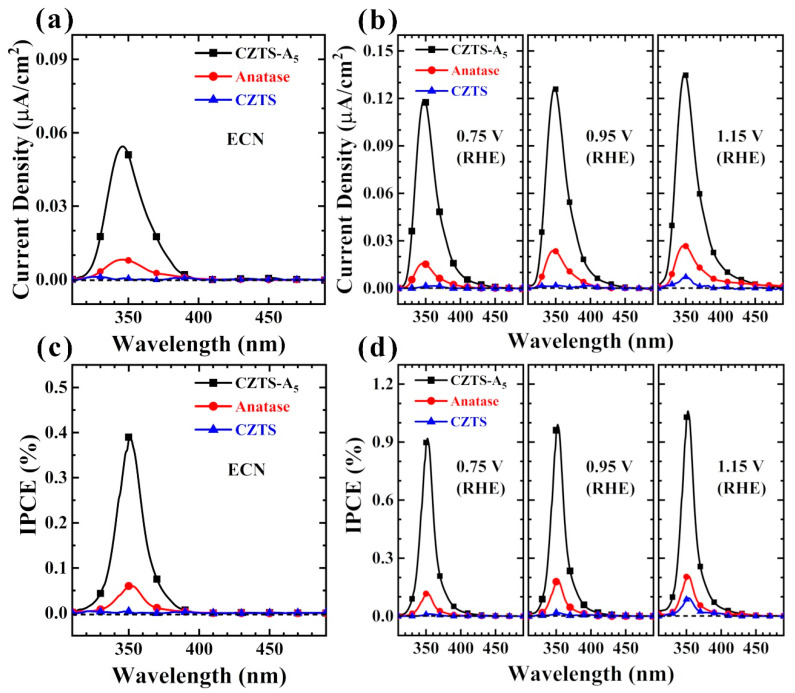
Relationship between photocurrent density and monochromatic light in electrochemical noise (ECN) mode (**a**) and photocurrent density and monochromatic light to generate IPCE (%) spectra (**b**); spectra of the relationship between photocurrent density and IPCE (%) spectra evaluated at different wavelengths of monochromatic light by applying different bias voltages (**c**,**d**) (electrolyte is 0.5 M Na_2_SO_4_, pH = 6).

Before conducting the addition bias test on the catalyst, we employed the electrochemical noise (ECN) mode to measure the photocurrent density of the sample without any bias voltage and before any damage to the catalyst’s performance for performance evaluation. This non-destructive and in situ monitoring technology can study the self-generated chemical reactions on photoanodes (Figure 7a,c).

Based on the data of hydrogen production and LSV measurements, further studies were only conducted on CZTS, TiO_2_ (anatase), and CZTS–A5 in the following measurements. To further explore the influence of the light absorption range of the photoanode, we performed IPCE testing on the photoanode.

As shown in Figure 7, the luminous flux density of CZTS–A_5_ is higher than that of CZTS and TiO_2_ (anatase). Figure 7d shows the monochromatic radiation characteristics of CZTS, TiO_2_ (anatase), and CZTS–A_5_ at different bias voltages. The IPCE value of CZTS–A_5_ is the highest at a wavelength of 358 nm, reaching 1.08%. Compared with CZTS and TiO_2_, the IPCE values have significantly increased 10 times (1.08% vs. 0.10%) and 5 times (1.08% vs. 0.20%), respectively, indicating that CZTS–A_5_ responds significantly to light. The photocurrent response of CZTS–A_5_ increases with the increase in the bias voltage, and the same phenomenon is shown in the linear sweep voltammetry (LSV) curve results. Similarly, the data demonstrate that CZTS–A_5_ has improved visible light absorption, mainly distributed below 480 nm, while the two undoped comparative samples of CZTS and TiO_2_ (anatase) do not show this improvement.

**Figure 8:**
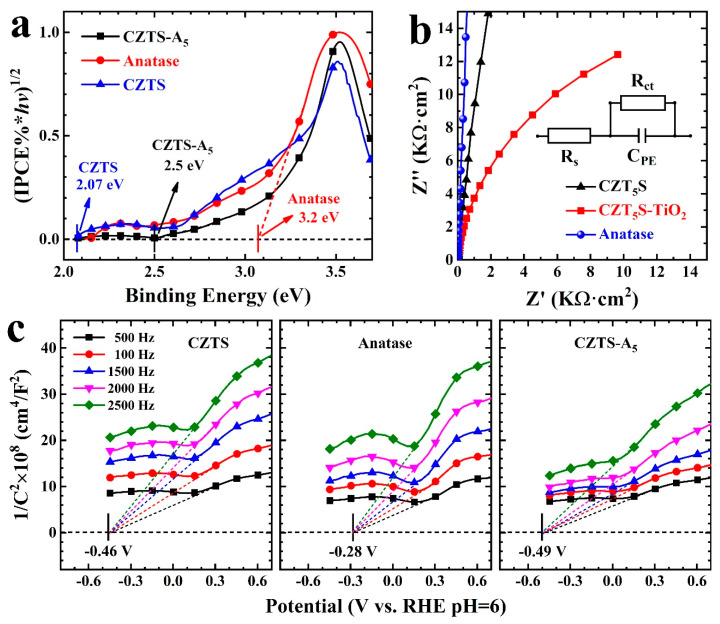
(**a**) Band gap plot determined by examining the relationship between (IPCE% × *hv*)^1/2^ and photon energy (*hv*); (**b**) EIS impedance plot; (**c**) Mott–Schottky plot.

The band gap (*E_g_*) of CZTS, CZTS–A_5_, and TiO_2_ (anatase) can be obtained from the IPCE spectra, as shown in Figure 8a, with values of 2.07 eV, 2.50 eV, and 3.20 eV, respectively. Thus, the band structure of the samples can be determined. To further verify the photoinduced e^−^/h^+^ separation, electrochemical impedance spectroscopy (EIS) was applied to the analysis of TiO_2_ (anatase), CZTS NPs, and CZTS nanocomposites (Figure 8b and Table S1). Typically, we believe that the radius of the arc is proportional to the electrochemical reaction rate of the catalyst on the electrode surface. The EIS diagram with the smallest arc radius has the highest electrochemical reaction rate, implying that it has the fastest interface charge transfer and the highest photoinduced e^−^/h^+^ separation efficiency. Compared with the mixed nanocomposites, the arc radius of TiO_2_ (anatase) is the largest, and its electrochemical reaction rate is the lowest. The samples CZTS and CZTS–A_5_ show Nyquist curves with gradually decreasing arc radii, indicating a higher efficiency of photoinduced e^−^/h^+^ separation. The arc radius of CZTS–A_5_ is the smallest, and the efficiency of photoinduced e^−^/h^+^ separation is the highest. Therefore, the EIS results confirm the results of the photocurrent response.

In addition, the band structure of the prepared samples was also studied using the Mott–Schottky measurement method. The calculated flat-band potentials of CZTS, TiO_2_ (anatase), and CZTS–A_5_ are −0.46 V, −0.28 V, and −0.49 V, respectively. Compared with CZTS and TiO_2_, the flat-band potential of CZTS–A_5_ moves more negatively (Figure 8c).

**Figure 9:**
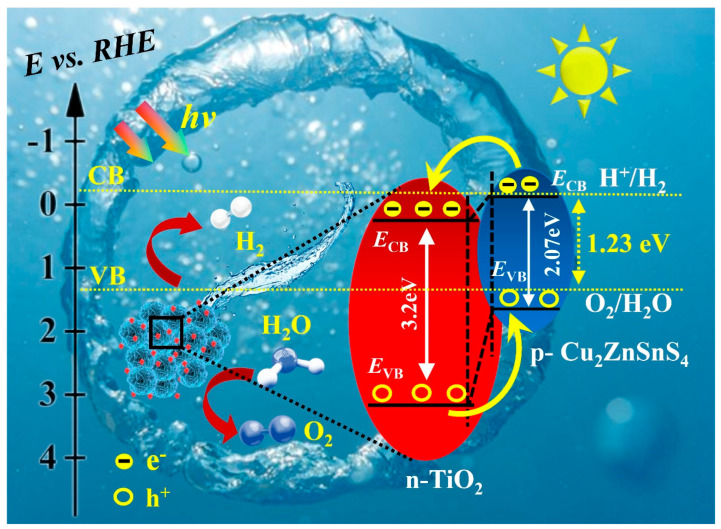
Proposed band gap structure diagram of the Cu_2_SnZnS_4_–TiO_2_ (anatase) hybrid nanocomposites.

In hydrogen production by the photocatalytic water decomposition catalyst, we propose a p-n heterojunctions mechanism, which is supported in subsequent experiments (see Figure 9). The significant improvement in photocatalytic hydrogen production efficiency is attributed to the strong enough built-in electric field formed by heterojunctions, which improves the efficiency of photo-generated charge carriers separation/transfer and enhances the production rate of photocatalytic H_2_ production. When CZTS–A_5_ is illuminated, electrons migrate from the conduction band (CB) of anatase titanium dioxide (TiO_2_) to the valence band (VB) of CZTS. Then, they are excited to the CB of CZTS, resulting in charge separation. The sulfur and oxygen vacancies generated in the CZTS–TiO_2_ hybrid nanocomposite facilitate the rapid migration of electrons and increase the photocatalytic activity. When electrons and holes are separated, they migrate towards CZTS and TiO_2_, respectively, which inhibits electron-hole recombination. In the whole mechanism of photocatalytic H_2_ generation, the oxidation reaction is driven by the holes in the VB of CZTS, and the holes consume ethanol to generate H^+^ ions and O_2_, which makes it easier to react with H^+^ ions, thereby increasing the generation rate of H_2_.

## 3. Materials and Methods

### 3.1. Materials

All the chemicals and reagents applied in this study were of analytical grade. CuCl_2_·2H_2_O (99%), Zn (CH_3_OO)_2_·2H_2_O (99%), SnCl_2_ (99%), thiourea (NH_2_CSNH_2_) (99%), anhydrous ethanol (C_2_H_6_O, AR), and tetra-n-butyl titanate (TBOT, C_16_H_36_O_4_Ti) were obtained from General-Reagent, Sinopharm Chemical Reagent Co., Ltd., Shanghai, China. All experiments were conducted using deionized water.

### 3.2. Synthesis Method

**Figure 10:**
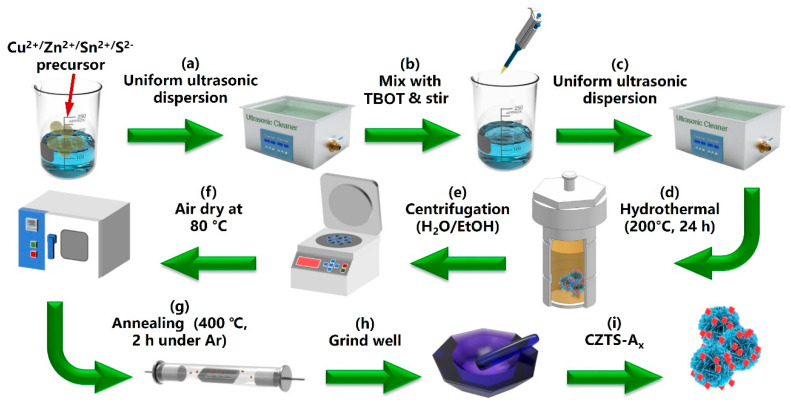
Schematic illustration for the fabrication strategy of the CZTS and the CZTS–A_x_ nanocomposite.

In the first step of the experiment, CuCl_2_·2H_2_O, Zn(CH_3_OO)_2_·2H_2_O, SnCl_2_, and thiourea in the ratio of (2:1:1:8), all with an average dosage of 1 mmol, were dissolved in a beaker containing a mixture of ultrapure water and anhydrous ethanol in a ratio of 1:1. The mixture was sonicated for 30 min at room temperature. After thorough stirring, tetra-n-butyl titanate (C_16_H_36_O_4_Ti, TBOT) was added dropwise in different ratios (0, 1, 3, 5, 7, 9 mL), and the mixture was sonicated for 1 h. The resulting suspension was transferred to a stainless-steel autoclave and heated at 200 °C for 24 h in an oven. The solution was then cooled, centrifuged, and washed repeatedly with deionized water and ethanol. The resulting black powder was homogenized and annealed at 400 °C under an argon atmosphere in a tube furnace for 2 h. The final sample was obtained after natural cooling. The experimental procedure is shown in Figure 10, and the final samples were denoted as CZTS–A_x_, where X = 0, 1, 3, 5, 7, or 9 mL of TBOT. Moreover, to evaluate whether the PC/PEC performances of the CZTS–A_x_ heterojunction hybrid nanocomposite were improved, pure Cu_2_ZnSnS_4_ (CZTS) NPs and pure TiO_2_ (anatase) NPs were prepared through a similar synthesis scheme.

### 3.3. Characterization

The crystal structure and phase of the composite materials were determined by X-ray diffraction using a X-ray diffractometer (Cu Kα1 1.5418 Å, 40 kV, 100 mA, measurement range of 20–80°, XRD, Rigaku D/max-2400, Akishima, Rigaku, Tokyo, Japan). Scanning electron microscopy equipped with energy dispersive spectroscopy (SEM, Apreo SLoVac, CZ, Thermo Fisher, Waltham, MA, USA) and high-resolution transmission electron microscopy (FEI-TALOS-F200X, Hillsboro, OR, USA) were used for the morphological analysis of the samples (TEM, HRTEM, SAED, Thermo Fisher Scientific, Waltham, MA, USA). Elemental stoichiometry was determined using an energy dispersive spectrometer (EDS) installed on the SEM (EDS, Apreo SLoVac, CZ, Thermo Fisher, Waltham, MA, USA). To evaluate surface functional groups, a Nicolet iS 50 Fourier transform infrared spectrometer (FT-IR, Thermo Fisher, Waltham, MA, USA) was used to perform Fourier-transform infrared spectroscopy (FT-IR) in the range of 400–4000 cm^−1^ on KBr pellets.Utilized X-ray photoelectron spectroscopy (XPS) with a monochromatic Al-K-α (1486.6 eV) radiation source (XPS, Thermo Fisher Scientific K–Alpha, Waltham, MA, USA) to investigate the elemental composition and electronic valence states of the composite materials. The specific surface area and porosity of the samples were characterized and confirmed using the Brunauer-Emmett-Teller (BET) method with the V-Sorb 2800P instrument (BET, Gold APP Instruments Corp. LTD, Beijing, China). Ultraviolet/visible diffuse reflectance spectra (UV-Vis) were recorded using a laboratory-equipped Cary 5000 spectrometer (UV-Vis, Agilent, Santa Clara, CA, USA), with BaSO_4_ as the reference. Electron paramagnetic resonance (EPR) spectra at room temperature were recorded using a Bruker (Billerica, MA, USA) A300 spectrometer to determine the oxygen vacancies and sulfur vacancies in the composite materials, (EPR, Bruker Biospin GmbH, Rheinstetten, Germany).

### 3.4. Photoelectrochemical (PEC) Measurements

In a specially designed experimental reactor, each photocatalyst was mixed with ultrapure water and naphthol. Then, the mixture was ultrasonicated for 30 min to achieve uniform dispersion. A tin-doped indium oxide (FTO) glass with an active area of 1 square centimeter was used as the substrate. A 50 µL suspension was uniformly dropped on the FTO glass and spin-coated six times. Then, the sample was air-dried overnight. Photocurrent measurement was carried out under a standard three-electrode system, with the FTO glass loaded with the sample as the working electrode. The effective illuminated area was 1.0 square centimeter, and a platinum plate was used as the counter electrode. The reference electrode was a Ag/AgCl electrode, and the electrolyte solution was 0.5 mol L^−1^ Na_2_SO_4_. Under the irradiation of a high-uniformity integrated xenon light source (PLS-FX300HU, Beijing Perfect Light Source Company, Beijing, China) with AM 1.5G (100 mW cm^−^²), the samples were measured using an electrochemical workstation (CHI760E) by linear sweep voltammetry (LSV) and chronoamperometry (I-t). During the linear sweep voltammetry (LSV) measurement, a shutter with a frequency of 5 s^−1^ was used to cut off the light. For the chronoamperometry (I-t) measurement, alternate illumination was cut off with a shutter of 300 s^−1^ and performed at a potential of 1.0 V (compared to RHE at pH = 6). The catalyst area of the working electrode was completely immersed in the electrolyte. By applying the Nernst equation to Equation (1), the measured potential was converted to the reversible hydrogen electrode (RHE) scale according to the Nernst equation (Equation (3)) [6,10,11,15,43,44,45,46]:

(3) $$E_{RHE}=E_{Ag/AgCl}+0.0592\times pH+E_{Ag/AgCl}^{0}$$

where the $E_{Ag/AgCl}^{0}$ = 0.1976 V vs. Ag/AgCl at room temperature.

The incident photon-to-current efficiency (IPCE) was measured using a 300 W xenon lamp equipped with a grating monochromator (7ISU, SOFN Instruments Co., Ltd., Beijing, China) and a filter to eliminate higher-order diffraction. A potentiated instrument (CS350H, Wuhan KRITE Instrument Co., Ltd., Wuhan, China) was used to collect and process the signals and for electrochemical impedance spectroscopy (EIS) and Mott–Schottky (M-S) curve measurements.

The test setup included an electrochemical workstation (CS350H, CorrTest, Wuhan, China), a 300 W xenon light source (PLS-SXE300D, Perfect Light Source Co., Ltd., Beijing, China), and a grating monochromator (7ISU, SOFN Instruments Co., Ltd., Beijing, China). The grating monochromator was equipped with a filter to eliminate higher-order diffraction for measuring the incident photon-to-current efficiency (IPCE). The IPCE value was determined using the formula provided (Equation (4)) [6,10,11,15,43,45].

(4) $$IPCE(\%)=\frac{1240\times J_{p}}{\lambda \times I_{0}}\times 100\%$$

where IPCE represents the incident photon-to-current efficiency, *λ* represents the wavelength of the incident light, *I*_0_ represents the photocurrent, and *J_p_* represents the incident light intensity.

In the dark, electrochemical impedance spectroscopy (EIS) and Mott–Schottky (M-S) plots were measured using an electrochemical workstation (Squidstat Plus, IME, Tempe, AZ, USA). The working frequency range of the electrochemical analyzer is from 0.01 to 100,000 Hz, with a voltage increment of 0.005 V and an AC amplitude of 10 mV. In a typical M-S measurement, the test frequencies of the working electrode are 500, 1000, 1500, 2000, and 2500 Hz, respectively [44,46,47,48].

### 3.5. Photocatalytic (PC) Hydrogen Evaluation

The MCP-WS1000 photochemical workstation, produced by Perfect Light Source Technology Co., Ltd., Beijing, China, includes a 50 mL quartz reactor to evaluate the photocatalytic (PC) hydrogen evolution. This device is employed to assess the performance of photocatalytic hydrogen production under full-spectrum light irradiation. In this configuration, the prepared photocatalyst is dispersed in a solution composed of anhydrous ethanol and an equal amount of ultrapure water by using ultrasound for 30 min. Before measuring the H_2_ evolution, it is purged with argon for 10 min. A lamp array consisting of 9 full-spectrum lamps (10 W) provides visible light, and a water-cooling system is utilized to maintain the solution temperature at 5 °C during the photocatalytic reaction. The hydrogen evolution generated per hour is analyzed using a GC9790II gas chromatograph system produced by Zhejiang Fuli Analytical Instruments Inc., Wenling, China.

## 4. Conclusions

This study successfully synthesized a CZTS–TiO_2_ (anatase) heterojunction nanocomposite using a one-step method, significantly enhancing the photocatalytic hydrogen production performance under solar irradiation. The nanocomposite’s small particle size, large specific surface area, and high conductivity were critical factors in improving its performance. Notably, the H_2_ yield of the CZTS–A_5_ nanocomposite reached 0.5017 mmol/g/h, 250-times higher than that of the original CZTS. This remarkable increase can be attributed to the unique p-n heterojunctions formed between the CZTS photocatalyst and TiO_2_ (anatase) loading. This structure benefits from the close interface contact between TiO_2_ and CZTS, increasing sulfur vacancies at the interface, enhancing the photocatalytic activity, and providing numerous active sites for the H_2_ generation reaction. Additionally, the earth-abundant nature of this nanocomposite’s elements ensures good reproducibility and low cost, highlighting its potential for large-scale application in photocatalytic processes. The outcomes of this study pave the way for new methods of inefficient photocatalytic H_2_ production and contribute to the advancement of sustainable solar technology for hydrogen fuel production.

## Figures and Tables

**Table 1:** Comparison of different pore size distributions of BET in CZTS NPs, TiO_2_ (anatase) NPs, and hybrid CZTS–A_X_ (X = 3, 5, 9) nanocomposites.

Samples	S_BET_ (m^2^ g^−1^)	Pore Volume (cm^3^ g^−1^)	Average Pore Size (nm)	Most Frequent Pore Diameter (nm)
anatase	87.223	0.372	1.805	–
CZTS	22.022	0.042	8.488	–
CZTS–A_3_	132.280	0.301	9.101	8.743
CZTS–A_5_	98.954	0.312	1.239	10.300
CZTS–A_9_	126.612	0.306	9.562	8.739

## Data Availability

The original contributions presented in the study are included in the article/Supplementary Material, further inquiries can be directed to the corresponding author/s.

## Supplementary Materials

The following supporting information can be downloaded at: https://www.mdpi.com/article/10.3390/molecules29112514/s1, Figure S1: SEM and EDS images of CZTS. Figure S2: SEM images of CZTS (a), CZTS–A_1_ (b), CZTS–A_3_ (c), CZTS–A_7_ (d), CZTS–A_9_ (e), and anatase (f). Figure S3: XPS full spectrum of CZTS and CZTS–A_5_ nanocomposites. Figure S4: The band gap diagram of CZTS, anatase, and CZTS–A_5_. Figure S5: Chopped linear sweep voltammetry (LSV) curve plot of the hybrid CZTS–A_x_ (x = 1, 3, 5, 7, 9) nanocomposites and TiO_2_ observed with a bias voltage of 1.5 V vs. RHE (electrolyte is 0.5 M Na_2_SO_4_, pH = 6). Figure S6. N_2_-adsorption desorption isotherms of CZTS NPs, hybrid CZTS-A_x_ (x = 3, 5, 9) nanocomposites, and TiO_2_ (Anatase). Table S1: The fitting results of the EIS measurement results.
