# Supplementary material for: A Convenient In Situ Preparation of Cu2ZnSnS4–Anatase Hybrid Nanocomposite for Photocatalysis/Photoelectrochemical Water-Splitting Hydrogen Production
Source: Molecules. 2024 May 26;29(11):2514. doi: 10.3390/molecules29112514 (PMC11173519; doi:10.3390/molecules29112514)
Supplement: Supplementary file 1 [file molecules-29-02514-s001.zip › molecules-3028335-supplementary.pdf]

Supporting Information

A Convenient In Situ Preparation of Cu_2ZnSnS_4–Anatase Hybrid Nanocomposite for Photocatalysis/Photoelectrochemical Water-Splitting Hydrogen Production

Ke-Xian Li [1,2,3], Cai-Hong Li [1,3,4], Hao-Yan Shi [1,3], Rui Chen [1,3], Ao-Sheng She [1,3], Yang Yang [1,2,3], Xia Jiang [1,3], Yan-Xin Chen [1,2,3,*] and Can-Zhong Lu [1,2,3,*]

[1] State Key Laboratory of Structural Chemistry, Fujian Science & Technology Innovation Laboratory for Optoelectronic Information of China, Fujian Institute of Research on the Structure of Matter, Chinese Academy of Sciences, Fuzhou 350108, China; xmlikexian@fjirsm.ac.cn (K.-X.L.); xmlicaihong@fjirsm.ac.cn (C.-H.L.); xmshihaoyan@fjirsm.ac.cn (H.-Y.S.); xmchenrui@fjirsm.ac.cn (R.C.); xmsheaosheng@fjirsm.ac.cn (A.-S.S.); xmyangyang@fjirsm.ac.cn (Y.Y.); xmjiangxia@fjirsm.ac.cn (X.J.)

[2] College of Chemistry and Materials Science, Fujian Normal University, Fuzhou 350108, China

[3] Xiamen Key Laboratory of Rare Earth Photoelectric Functional Materials, Xiamen Institute of Rare-Earth Materials, Haixi Institutes, Chinese Academy of Sciences, Xiamen 361021, China

[4] College of Chemistry, Fuzhou University, Fuzhou 350116, China

* Correspondence: yanxinchen@fjirsm.ac.cn (Y.-X.C.); czlu@fjirsm.ac.cn (C.-Z.L.)

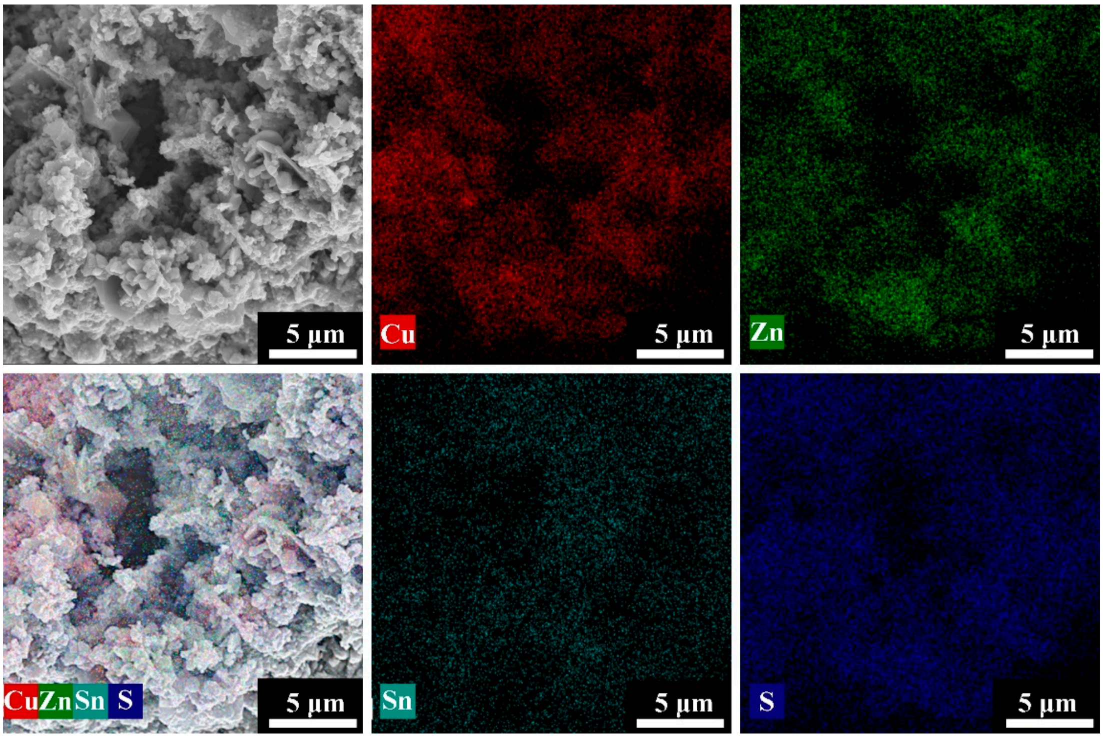

Figure S1. SEM and EDS images of CZTS.

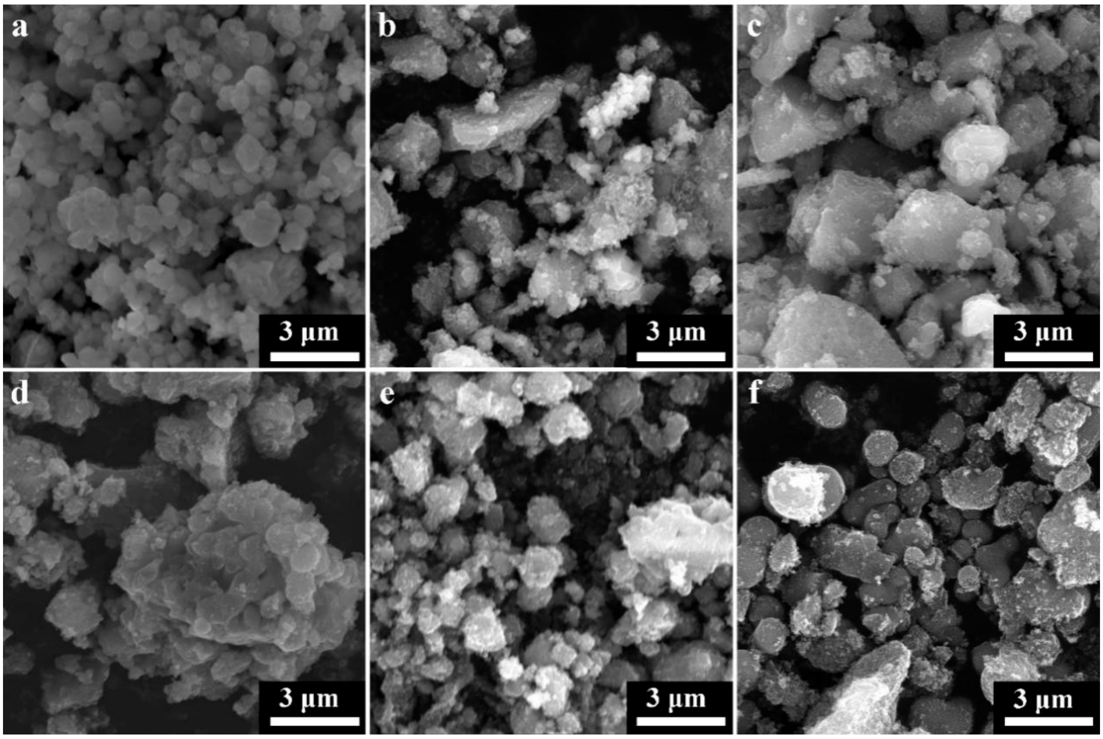

Figure S2. SEM images of CZTS (a), CZTS-A_1 (b), CZTS-A_3 (c), CZTS-A_7 (d), CZTS-A_9 (e) and Anatase (f).

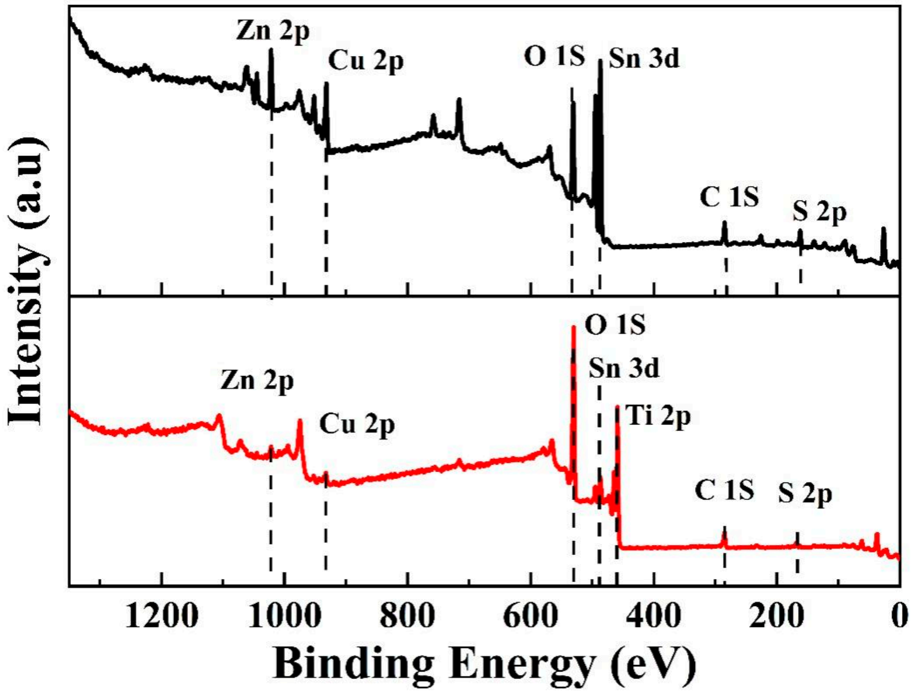

Figure S3. XPS full spectrum of CZTS and CZTS-A5 nanocomposites.

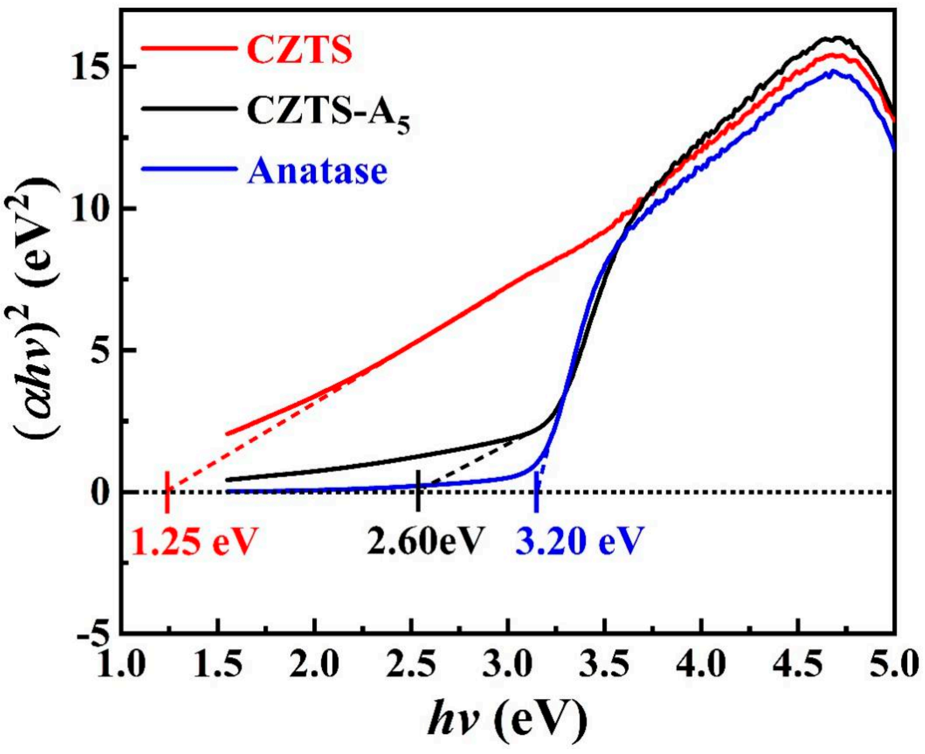

Figure S4. The bandgap diagram of CZTS, Anatase and CZTS-A5.

This paper uses Ultraviolet Photoelectron Spectroscopy (UPS) as an additional method to verify the bandgap structure diagram. As seen from **Figure 6b**, the color of the sample changes from dark black to pure white. Due to the limitations of our instrumentation, when UPS is used to test black powder samples, the UV absorption peak of the black sample shows complete absorption. The existence of such errors makes Ultraviolet Photoelectron Spectroscopy (UPS) a method to confirm the bandgap structure diagram to corroborate the bandgap structure from the side in this paper.

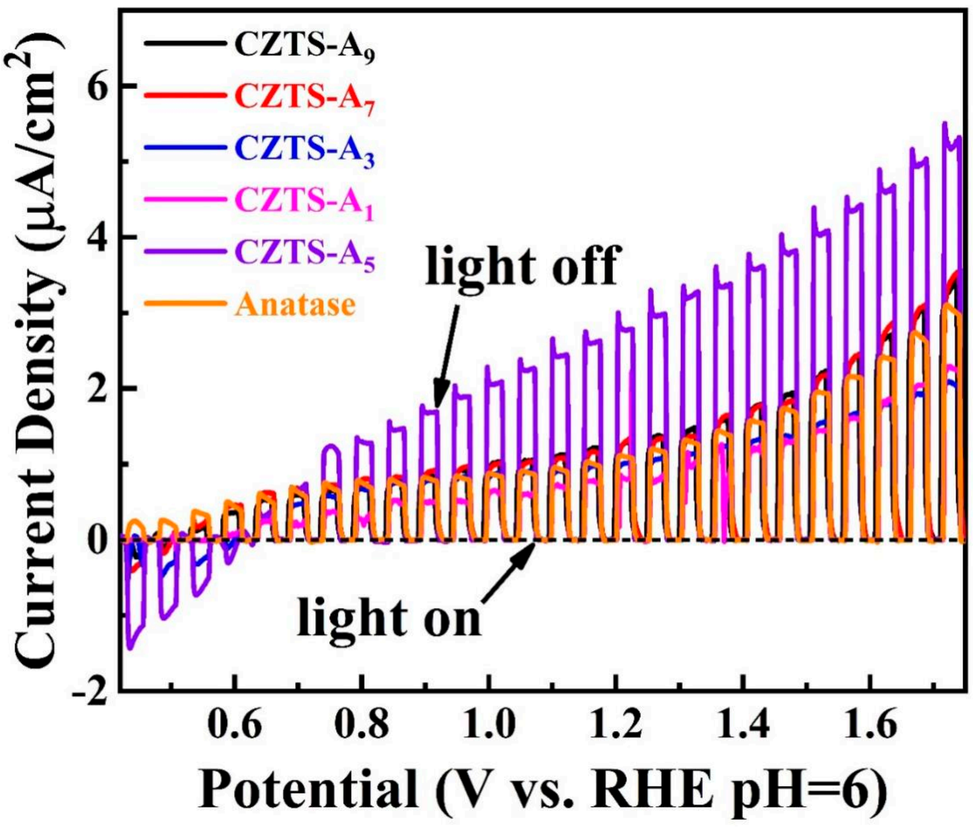

Figure S5. Chopped linear sweep voltammetry (LSV) curve plot of the hybrid CZTS-A$_x$ (x=1,3,5,7,9) nanocomposites and TiO_2 observed with a bias voltage of 1.5V vs. RHE (electrolyte is 0.5 M Na_2SO_4, pH = 6).

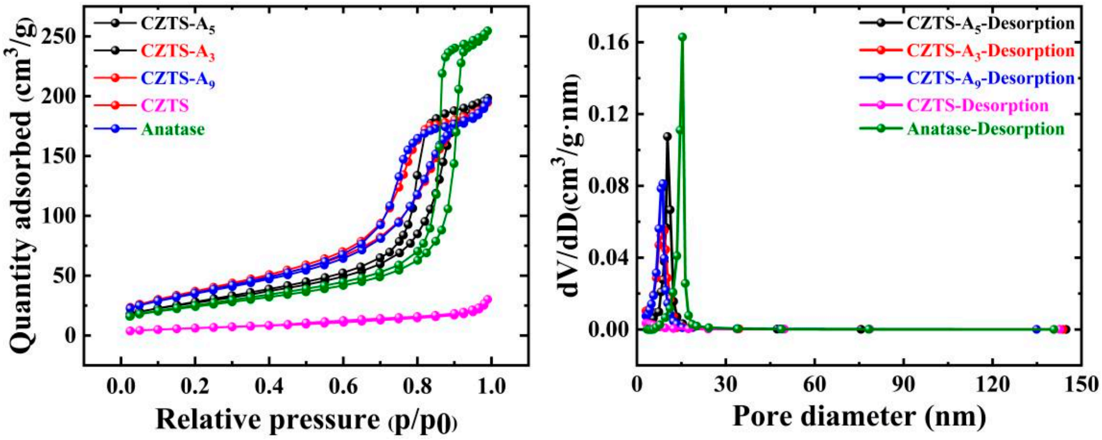

Figure S6. N_2-adsorption desorption isotherms of CZTS NPs, hybrid CZTS-A_x (x=3, 5, 9) nanocomposites, and TiO_2 (Anatase).

Table S1. The fitting results of the EIS measurement results.

samples	Rs(Ω)	Rct(KΩ)
TiO_2	9.02	1760
CZTS	12.8	1400
CZTS-A_5	9.93	30.7
